# P-1457. Association of Food Insecurity Risk with HIV Viral Non-suppression among People with HIV, Accessing Care in an Urban Infectious Disease Clinic in Baltimore, MD

**DOI:** 10.1093/ofid/ofae631.1629

**Published:** 2025-01-29

**Authors:** Tarfa I Verinumbe, Ye Eun Lee, Tracy Agee, Heidi Hutton, Sharon Kelly, Jeffrey H Hsu, Joyce L Jones, Sheree R Schwartz, Oluwaseun Falade-Nwulia

**Affiliations:** Johns Hopkins University, Baltimore, Maryland; Johns Hopkins University School of Medicine, Baltimore, Maryland; Johns Hopkins University, Baltimore, Maryland; Johns Hopkins University School of Medicine, Baltimore, Maryland; Johns Hopkins University School of Medicine, Baltimore, Maryland; Johns Hopkins University School of Medicine, Baltimore, Maryland; Johns Hopkins University School of Maryland, Baltimore, Maryland; Johns Hopkins School of Public Health, Baltimore, Maryland; Johns Hopkins University, Baltimore, Maryland

## Abstract

**Background:**

Food insecurity disproportionately affects people with HIV (PWH) and is associated with increased HIV transmission, interruptions in HIV care, and poor medication adherence. The intersection of depression, anxiety, substance use, and food insecurity among PWH may further exacerbate adverse HIV outcomes. This study assesses the association of food insecurity risk with viral load (VL) non-suppression among PWH, with or at risk for comorbid mental health (MHD) or substance use disorder (SUD), in HIV care.

**Figure:**
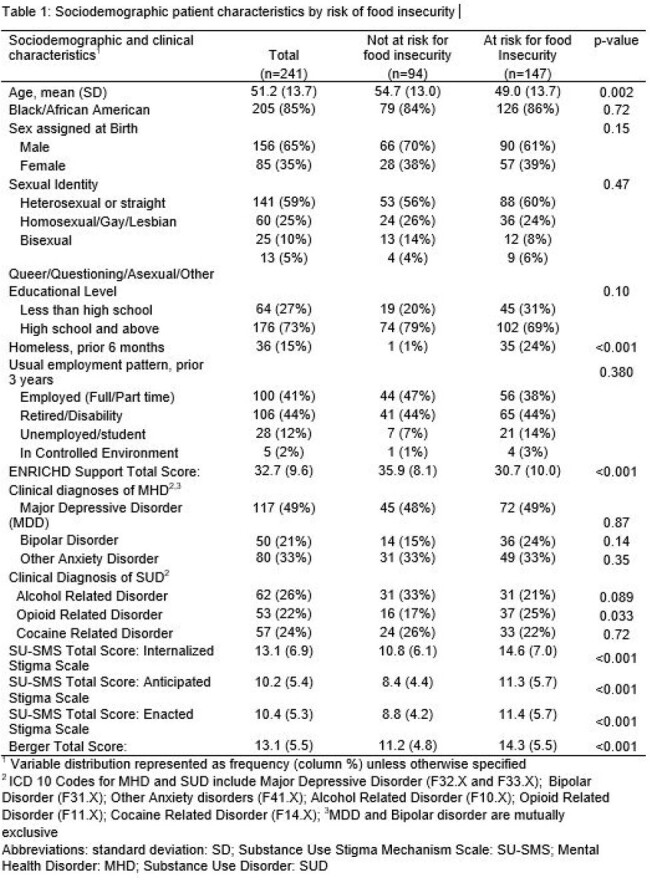

**Methods:**

PWH in care at The Johns Hopkins Bartlett Infectious Disease Clinic who screened positive for depression (PHQ-2), anxiety (GAD-2), or substance use (NIDA Quick Screen), were enrolled in an interventional trial evaluating optimal strategies for engaging PWH in MHD and SUD treatment. Food insecurity risk was assessed at baseline using a 2-item validated tool: The Hunger Vital Sign. Patients were considered at risk if they reported a response other than “never” to either question on the tool: 1) worry about running out of food or 2) having insufficient food in the prior 12 months. HIV VL assay performed as part of routine care within 6 months of study enrollment were used to evaluate for VL non-suppression. The association of food insecurity risk with VL non-suppression (HIV RNA > 200 copies/ml) was assessed using log-binomial regression adjusting for age, sex, race, education, employment, homelessness, and social support.

**Results:**

Among 241 participants, mean age was 51 years (SD 13.7), 85% were Black, 65% were male, 74% had a clinical diagnosis of MHD and 44% a diagnosis of SUD. Most participants were employed (41%) or retired/on disability (44%). Homelessness in the prior 6 months was reported by 15%. Overall, 61% had food insecurity risk and 13% were virally non-suppressed. PWH at risk of food insecurity were more likely to be virally non-suppressed compared to food secure individuals (adjusted PR: 3.16; 95% CI: 1.26 – 7.91).

**Conclusion:**

Food insecurity risk was high in this study of PWH with or at risk for MHD/SUD, and was significantly associated with VL non-suppression. In populations of PWH with or at risk for MHD/SUD, screening for food insecurity as part of routine HIV care and subsequent linkage of individuals at risk to food intervention programs may support improved VL suppression.

**Disclosures:**

**Oluwaseun Falade-Nwulia, MBBS ,MPH**, Abbvie Inc: Grant/Research Support|Gilead Sciences: Advisor/Consultant

